# Efficient Implementation of Discrete-Time Quantum Walks on Quantum Computers

**DOI:** 10.3390/e26040313

**Published:** 2024-04-02

**Authors:** Luca Razzoli, Gabriele Cenedese, Maria Bondani, Giuliano Benenti

**Affiliations:** 1 Center for Nonlinear and Complex Systems, Dipartimento di Scienza e Alta Tecnologia, Università degli Studi dell’Insubria, Via Valleggio 11, 22100 Como, Italy; gcenedese@uninsubria.it (G.C.); giuliano.benenti@uninsubria.it (G.B.); 2 Istituto Nazionale di Fisica Nucleare, Sezione di Milano, Via Celoria 16, 20133 Milano, Italy; 3 Istituto di Fotonica e Nanotecnologie, Consiglio Nazionale delle Ricerche, Via Valleggio 11, 22100 Como, Italy; maria.bondani@cnr.it; 4 National Enterprise for nanoScience and nanoTechnology, Istituto Nanoscienze-CNR, Piazza San Silvestro 12, 56127 Pisa, Italy

**Keywords:** quantum walks, quantum computing, quantum circuits, quantum algorithms

## Abstract

Quantum walks have proven to be a universal model for quantum computation and to provide speed-up in certain quantum algorithms. The discrete-time quantum walk (DTQW) model, among others, is one of the most suitable candidates for circuit implementation due to its discrete nature. Current implementations, however, are usually characterized by quantum circuits of large size and depth, which leads to a higher computational cost and severely limits the number of time steps that can be reliably implemented on current quantum computers. In this work, we propose an efficient and scalable quantum circuit implementing the DTQW on the $2^{n}$-cycle based on the diagonalization of the conditional shift operator. For *t* time steps of the DTQW, the proposed circuit requires only $O(n^{2}+nt)$ two-qubit gates compared to the $O(n^{2}t)$ of the current most efficient implementation based on quantum Fourier transforms. We test the proposed circuit on an IBM quantum device for a Hadamard DTQW on the 4-cycle and 8-cycle characterized by periodic dynamics and by recurrent generation of maximally entangled single-particle states. Experimental results are meaningful well beyond the regime of few time steps, paving the way for reliable implementation and use on quantum computers.

## 1. Introduction

Quantum walks were formally introduced in the 1990s as the quantum analogue of classical random walks [1,2], but the seminal concept dates back to Feynman’s checkerboard (see Problem 2–6 in [3]), which connects the spin with the zig-zag propagation of a particle spreading at the speed of light across a (1 + 1)-dimensional spacetime lattice. The great potential of exploiting the peculiar features of quantum walks—quantum superposition of multiple paths, ballistic spread (faster than the diffusive spread of a classical random walker), and entanglement—for algorithmic purposes [4,5,6] has been immediately clear since their introduction. Nowadays, quantum walks have proven to be a universal model for quantum computation [7,8,9,10,11], and examples of their use include algorithms for quantum search [12,13,14,15]; the solving of hard *K*-SAT instances [16]; graph isomorphism problems [17,18,19]; algorithms in complex networks [20,21] such as link prediction [22,23] and community detection [24,25];  and quantum simulation [26,27,28,29,30]. Furthermore, quantum communication protocols based on quantum walks have been put forward [31,32,33,34,35,36,37,38,39]. For comprehensive reviews on quantum walks and their applications, we refer the reader to [40,41,42] and to [43,44,45] for the their physical implementations.

There are two main models of the quantum walk: the discrete-time quantum walk (DTQW) [1] and the continuous-time quantum walk (CTQW) [2]. The CTQW is defined on the position Hilbert space of the quantum walker, and the evolution is driven by the Hamiltonian *H* of the system, U(t)≡exp(−itH/ℏ). The DTQW is defined on a Hilbert space comprising an additional coin space, and the evolution is driven by a position shift operator, *S*, controlled by a quantum coin operator, *C*, acting at discrete time steps. The single time-step operator is defined as $U=S(C⊗I_{p})$, where $I_{p}$ is the identity in position space, from which $U\left(t\right)\equiv U^{t}$ with t∈N. As pointed out in [46], implementing the evolution operator of a DTQW is simplified by the fact that (i) the time is discrete, (ii) the evolution is repetitive, $U\left(t\right)=U^{t}$, and (iii) *U* acts locally on the coin–vertex states encoding the graph (applying *U* to the coin–vertex states associated with a given vertex will propagate the corresponding amplitudes only to adjacent vertices).

DTQWs already have efficient physical implementations in platforms that natively support the conditional walk operations, e.g., photonic systems [44,45]. However, devising efficient implementations on digitized quantum computers is desirable and necessary in order to make DTQWs available to develop quantum algorithms for general purpose quantum computers and, in general, quantum protocols to be implemented in circuit models. A first circuit implementation of a DTQW on the cycle was realized on a multiqubit nuclear magnetic resonance system [47], and thereafter, proposals for efficient implementation on certain graphs [48,49,50,51], for position-dependent coin operators [52], and for staggered quantum walks (a coinless discrete-time model of quantum walk) [53] have been devised.

In this work, we propose an efficient and scalable quantum circuit implementing the DTQW on the $2^{n}$-cycle, a finite discrete line with $2^{n}$ vertices and periodic boundary conditions. Although this is the simplest DTQW one may think of, implementing it on quantum computers already highlights the limitations of actual quantum devices [54,55,56,57]. In this model, the position state of the quantum walker is encoded in an *n*-qubit state. To the best of our knowledge, the most efficient state-of-the-art implementation of a DTQW [58] overall requires $O(n^{2}t)$ two-qubit gates for *t* time steps, because it involves one quantum Fourier transform (QFT) and one inverse QFT (IQFT) at *each* time step. In quantum computers, two-qubit gates are the noisiest and take the longest time to execute, so any efficient quantum circuit should aim at significantly reducing their number. Our quantum circuit accomplishes this task through a wise use of the unitary property of the QFT: independent of *t*, our circuit involves only one QFT (at the beginning) and one IQFT (at the end), and thus, it overall requires $O(n^{2}+nt)$ two-qubit gates. Accordingly, the advantage becomes larger and larger for long times, passing, for t≫n, from $O(n^{2}t)$ in [58] to O(nt) in the present scheme. For illustrative purposes, we implemented the proposed quantum circuit on actual quantum hardware—ibm_cairo v.1.3.5, a 27-qubit high-fidelity quantum computer—considering a Hadamard DTQW on the 4- and 8-cycles. Results indicate that our circuit outperforms current efficient circuits also in the regime of few time steps and provides experimental evidence of the recurrent generation of maximally entangled single-particle states in the 4-cycle [59].

This paper is organized as follows. Section 2 reviews the DTQW model on the *N*-cycle. Section 3 introduces the efficient quantum circuit we designed for the DTQW on the $2^{n}$-cycle and compares it with other existing schemes. Section 4 presents and discusses the results from testing the proposed circuit on quantum hardware. Finally, Section 5 is devoted to conclusions and perspectives. Selected technical details are deferred to the Appendices.

## 2. The Model: DTQW on the *N*-Cycle

**Figure 1:**
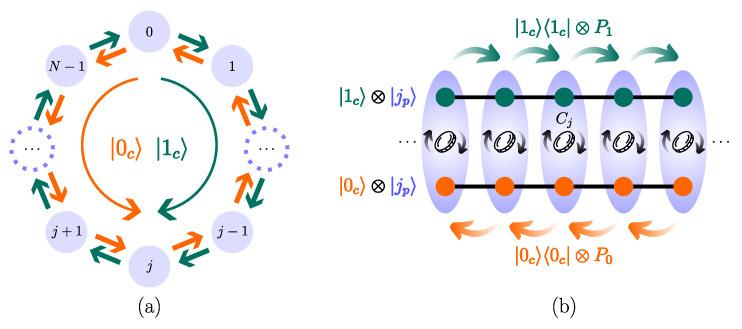
Schematic representation of a DTQW on the *N*-cycle. (**a**) The coin state (internal degree of freedom) is responsible for making the walker move in the cycle clockwise if $|1_{c}\rangle$ and counterclockwise if $|0_{c}\rangle$. (**b**) States and operators of a DTQW. The vertices of the cycle (light violet)—walker’s position states—are labeled by $|j_{p}\rangle$ with j=0,1,…,N−1, and each vertex comprises two subvertices—coin states—labeled by $|0_{c}\rangle$ (orange) and $|1_{c}\rangle$ (green). Each step of the walk, Equation (2), involves the action of a local coin operator $C_{j}$ responsible for mixing the coin states of each vertex (we assume $C_{j}=C$∀j), followed by the action of the conditional-shift operators $|0_{c}\rangle \langle 0_{c}|⊗P_{0}$ (decrement) and $|1_{c}\rangle \langle 1_{c}|⊗P_{1}$ (increment) responsible for shifting the position states, see Equation (3) [43].

An *N*-cycle, or circle, is a 1D lattice having *N* vertices and periodic boundary conditions. To each vertex, labeled by j=0,…,N−1, we associate a quantum state, |j〉, which represents the walker localized at such a vertex. In a DTQW, the quantum walker has an *external* degree of freedom, the position, and an *internal* one, the coin. Associated with each degree of freedom is a Hilbert space: an *N*-dimensional position Hilbert space $H_{p}^{\left(N\right)}=\mathrm{span}\left(\{|j_{p}\rangle :j=0,\ldots ,N-1\}\right)$ and a two-dimensional coin Hilbert space $H_{c}^{\left(2\right)}=\mathrm{span}\left(\{|s_{c}\rangle :s=0,1\}\right)$. We use the label “p” to refer to the walker’s position degree of freedom and “c” to the coin degree of freedom. Depending on the coin state, the walker can move counterclockwise (s=0) or clockwise (s=1) on the cycle (Figure 1). The full Hilbert space $H\equiv H_{c}^{\left(2\right)}⊗H_{p}^{\left(N\right)}$ is

(1) $$H=\mathrm{span}\left(\{|s_{c}\rangle |j_{p}\rangle :s=0,1;j=0,\ldots ,N-1\}\right).$$

This is the natural basis for a DTQW, and in the following, we will refer to it as the computational basis. The coin basis states are $|0_{c}{\rangle =\left(1,0\right)}^{⊺}$ and $|1_{c}{\rangle =\left(0,1\right)}^{⊺}$, with ^⊺^ denoting the transpose without complex conjugation; the position basis states are $|j_{p}{\rangle =\left(0,\ldots ,0,1,0,\ldots ,0\right)}^{⊺}$, with the only nonzero element in position *j*. Accordingly, a generic coin–position basis state $|s_{c}\rangle |j_{p}\rangle =|s_{c}\rangle ⊗|j_{p}\rangle$ is represented by the column vector of length 2N, whose first *N* entries are related to s=0 and the last *N* to s=1. The only nonzero entry is the (Ns+j)-th one.

**Figure 2:**
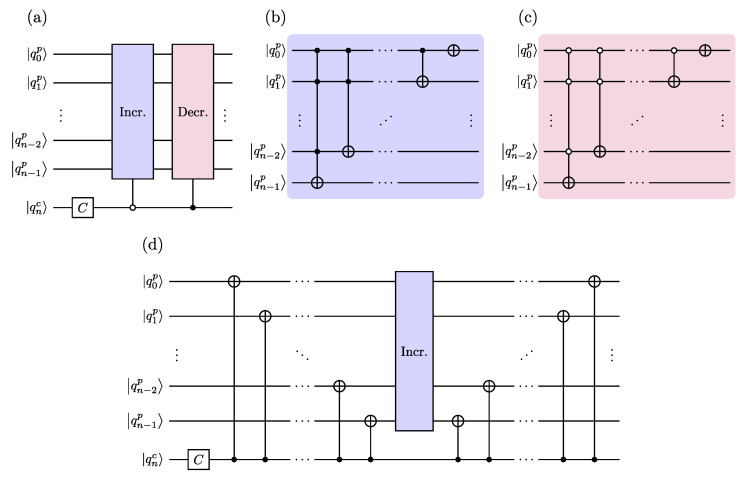
(**a**) Quantum circuit implementing one time step of the DTQW on the $2^{n}$-cycle based on controlled-*increment* (I) and controlled-*decrement* (D) gates [48]. (**b**) Increment and (**c**) decrement gates consist of generalized CNOT gates, with controls being |1〉 (solid circle) and |0〉 (empty circle), respectively. These gates act on the walker’s position quantum register, conditional on the coin’s qubit state (see panel (**a**)). (**d**) The ID-quantum circuit shown in panel (**a**) can be conveniently re-designed in terms of one increment gate (not controlled by the coin qubit) and CNOT gates only, being $\mathrm{Decr}.=\left({⨂}_{k=1}^{n}X_{k}\right)\mathrm{Incr}.\left({⨂}_{k=1}^{n}X_{k}\right)$ [58]. Quantum circuits in panels (**a**,**d**) implement the conditional shift operator $S=\sum_{j=0}^{N-1}(|0_{c}\rangle \langle 0_{c}\left|⊗\right|{\left(j+1\;\mathrm{mod}\;N\right)}_{p}\rangle \langle j_{p}\left|+\right|1_{c}\rangle \langle 1_{c}\left|⊗\right|{\left(j-1\;\mathrm{mod}\;N\right)}_{p}\rangle \langle j_{p}|)$, having the opposite convention to (3) used in the present work.

The evolution is ruled by the unitary single time-step operator

(2) $$U=S(C⊗I_{p}),$$

where $I_{p}$ is the identity in position space and *C* the coin operator acting on the coin state. Coin and conditional shift operators must be unitary for *U* to be unitary. The conditional shift operator *S* acts on the full Hilbert space and makes the walker move according to the coin state: $S|s_{c}\rangle |j_{p}\rangle =|s_{c}{\rangle |\left[\left(j+2s-1\right)\;\mathrm{mod}\;N\right]}_{p}\rangle$, where operations in position space are performed modulo *N*. Such an operator can be written as

(3) $$S=\sum_{s=0}^{1}\sum_{j=0}^{N-1}|s_{c}\rangle \langle s_{c}{|⊗|\left[\left(j+2s-1\right)\;\mathrm{mod}\;N\right]}_{p}\rangle \langle j_{p}|\equiv \sum_{s=0}^{1}|s_{c}\rangle \langle s_{c}|⊗P_{s},$$

where $P_{s}|j_{p}{\rangle =|\left[\left(j+2s-1\right)\;\mathrm{mod}\;N\right]}_{p}\rangle$. The operators $P_{0}$ (decrement) and $P_{1}$ (increment) are responsible for making the walker move one step counterclockwise and clockwise, respectively, (see Figure 1 and Figure 2b,c). In the computational basis, the conditional shift operator (3) has the matrix representation

(4) $$S=\left(\begin{array}{cc} P_{0} & 0\\ 0 & P_{1} \end{array}\right),$$

where 0 is the N×N null matrix and

(5) $$P_{0}=\left(\begin{array}{ccccc} 0 & 1 & 0 & \ldots & 0\\ 0 & \ddots & \ddots & \ddots & \vdots \\ 0 & \ddots & \ddots & 1 & 0\\ \vdots & \ddots & 0 & 0 & 1\\ 1 & \cdots & 0 & 0 & 0 \end{array}\right),\;P_{1}=\left(\begin{array}{ccccc} 0 & 0 & 0 & \ldots & 1\\ 1 & \ddots & \ddots & \ddots & \vdots \\ 0 & \ddots & \ddots & 0 & 0\\ \vdots & \ddots & 1 & 0 & 0\\ 0 & \cdots & 0 & 1 & 0 \end{array}\right)=P_{0}^{⊺}.$$

The quantum walker is usually assumed to be initially localized at the vertex |0〉, while the coin is in a generic superposition state,

(6) $$|\psi_{0}\rangle =\left[\cos\left(\frac{\theta }{2}\right)|0_{c}\rangle +e^{i\phi }\sin\left(\frac{\theta }{2}\right)|1_{c}\rangle \right]|0_{p}\rangle ,$$

with θ∈[0,π] and ϕ∈[0,2π[. After t∈N time steps, the quantum walker will be in the state

(7) $$|\psi_{t}\rangle =U^{t}|\psi_{0}\rangle =\sum_{j=0}^{N-1}\left[\psi_{0,j}\left(t\right)|0_{c}\rangle |j_{p}\rangle +\psi_{1,j}\left(t\right)|1_{c}\rangle |j_{p}\rangle \right],$$

where the amplitudes $\psi_{s,j}\left(t\right)\in C$—with s=0,1—associated with the states $|s_{c}\rangle |j_{p}\rangle$ satisfy the normalization condition at any *t*, $\sum_{j=0}^{N-1}\sum_{s=0}^{1}{\left|\psi_{s,j}\left(t\right)\right|}^{2}=1$. The probability to find the walker at position *k* at time *t*, irrespective of the coin state, is $p_{k}\left(t\right)=\sum_{s=0}^{1}{\left|\psi_{s,k}\left(t\right)\right|}^{2}$.

## 3. Quantum Circuit Implementing the DTQW on the $2^{n}$-Cycle

A quantum circuit implementing the DTQW on the *N*-cycle with $N=2^{n}$ requires n+1 qubits: *n* to encode the walker’s position state and an additional 1 to encode the coin state. Both states are encoded in base 2. Denoting by $j_{2}$ the binary representation of the integer *j* with *n* digits, in the *little-endian* ordering convention (the most significant bit is placed on the left), we write

(8) $$\left|j\rangle \equiv \right|j_{2}\rangle =|q_{n-1}\ldots q_{0}\rangle ,$$

where $q_{k}=0,1$ with k=0,…,n−1, such that $j=\sum_{k=0}^{n-1}q_{k}\times 2^{k}$. Accordingly, we write the quantum state of the quantum walker as the (n+1)-qubit state

(9) $$|s_{c}\rangle |j_{p}\rangle \equiv |q_{n}^{c}q_{n-1}^{p}\ldots q_{0}^{p}\rangle =|q_{n}^{c}\rangle ⊗|q_{n-1}^{p}\rangle ⊗\ldots ⊗|q_{0}^{p}\rangle ,$$

which represents the state where the coin is in the state $|q_{n}^{c}\rangle$ and the walker is in the position state $|q_{n-1}^{p}\ldots q_{0}^{p}\rangle$.

### 3.1. Quantum Circuit Design

The efficiency of a quantum circuit implementing a DTQW relies on the efficient implementation of the single time-step operator (2), so, ultimately, on that of the conditional shift operator (3). As shown in Equation (4), the latter involves the circulant matrices $P_{0}$ and $P_{1}$ introduced in Equation (5), and circulant matrices are known to be diagonalized by the quantum Fourier transform (QFT) matrix (see Appendix A). The QFT of the computational basis is defined as

(10) $$\mathrm{QFT}:\;|j\rangle \;\mapsto \;\frac{1}{\sqrt{N}}\sum_{k=0}^{N-1}\omega_{N}^{jk}|k\rangle ,$$

where $\omega_{N}=\exp\left(2\pi i/N\right)$ and j,k=0,…,N−1, with matrix representation $F_{j,k}=\omega_{N}^{jk}/\sqrt{N}$. The inverse QFT (IQFT) is represented by ${F^{†}}_{j,k}=\omega_{N}^{-jk}/\sqrt{N}$. The QFT is a unitary transformation, $FF^{†}=F^{†}F=I$. Accordingly, we can write

(11) $$P_{0}=F^{†}\Omega^{†}F,\;\mathrm{and}\;P_{1}=F^{†}\Omega F,$$

where

(12) $$\Omega =\mathrm{diag}\left(1,\omega_{N}^{1},\ldots ,\omega_{N}^{N-1}\right)=R_{1}⊗R_{2}⊗\cdots ⊗R_{n}=\overset{n}{\underset{k=1}{⨂}}R_{k},$$

with

(13) $$R_{k}=\left(\begin{array}{cc} 1 & 0\\ 0 & e^{\frac{2\pi i}{2^{k}}} \end{array}\right)=\left(\begin{array}{cc} 1 & 0\\ 0 & \omega_{N}^{2^{n-k}} \end{array}\right).$$

We stress that $\Omega |j_{p}\rangle =R_{1}|q_{n-1}^{p}\rangle ⊗\ldots R_{n}|q_{0}^{p}\rangle$ (order matters) and that we can write the second equality of Equation (12) because we are assuming $N=2^{n}$. Given Equation (11), the conditional shift matrix *S* (4) and its diagonal form Σ are related via

(14) $$\Sigma =\left(I_{c}⊗F\right)S\left(I_{c}⊗F^{†}\right)=|0_{c}\rangle \langle 0_{c}|⊗\Omega^{†}+|1_{c}\rangle \langle 1_{c}|⊗\Omega =\left(\begin{array}{cc} \Omega^{†} & 0\\ 0 & \Omega \end{array}\right),$$

where the identity in coin space $I_{c}$ is required since the (I)QFT acts on position space only.

**Figure 3:**
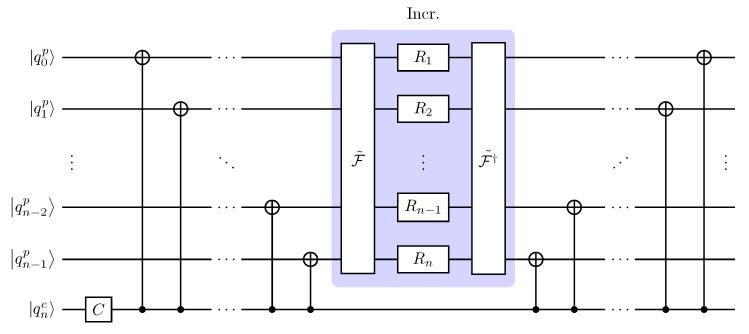
Quantum circuit implementing one time step of the DTQW on the $2^{n}$-cycle proposed in [58]. The quantum Fourier transform, $\tilde{F}$, and its inverse, ${\tilde{F}}^{†}$, do *not* include the SWAP gates. The increment gate is diagonalized by the QFT (see also Figure 2d). Conditional shift operator is as in Figure 2.

The DTQW of *t* steps is generated by repeatedly applying the operator *U* *t* times, Equation (7). Recalling that the QFT is unitary, it acts only on position space, and given that $S=\left(I_{c}⊗F^{†}\right)\Sigma \left(I_{c}⊗F\right)$ and that $\left(C⊗I_{p}\right)=\left(I_{c}⊗F^{†}\right)\left(C⊗I_{p}\right)\left(I_{c}⊗F\right)$, we can write

(15) $$\begin{array}{cc} U^{t} & =\left(I_{c}⊗F^{†}\right){\left[\Sigma (C⊗I_{p})\right]}^{t}\left(I_{c}⊗F\right), \end{array}$$

with Σ in Equation (14). Equation (15) provides a first sketch of the circuit we are going to implement. First, we perform a QFT on the position register, $I_{c}⊗F$. Then, we repeat *t* times the single time-step evolution in the extended Fourier space (extended to include coin space), $\Sigma (C⊗I_{p})$. In the end, we perform an IQFT on the position register, $I_{c}⊗F^{†}$. Even at this stage, an advantage of our scheme is evident: overall, it requires only one QFT and one IQFT, unlike the QFT scheme in Figure 3, which requires both the transformations at *each* time step [58].

Now, we focus on Σ to further improve the above scheme. The second equality in Equation (14) clearly shows the action of Σ: if the coin is in the state $|0_{c}\rangle$ ($|1_{c}\rangle$), then the operator $\Omega^{†}$ (Ω) acts on the position state. It is evident that, in the present form, each step of the DTQW requires 2n controlled-$R_{k}$ gates. To reduce the number of controlled operations, we point out that performing $R_{k}^{†}=\mathrm{diag}\left(1,\exp\left[-2\pi i/2^{k}\right]\right)$ on the *k*-th position qubit if the coin is in the state $|0_{c}\rangle$ and $R_{k}=\mathrm{diag}\left(1,\exp\left[2\pi i/2^{k}\right]\right)$ (opposite phase) if in $|1_{c}\rangle$ is equivalent to performing $R_{k}^{†}$ regardless of the coin state, followed by a controlled-$R_{k}^{2}$ if the coin is in $|1_{c}\rangle$ to compensate the previously assigned phase and obtain the correct one. Formally, we can rewrite the diagonal conditional shift operator (14) as

(16) $$\Sigma =(|0_{c}\rangle \langle 0_{c}|⊗I_{p}+|1_{c}\rangle \langle 1_{c}|⊗\Omega^{2})\left(I_{c}⊗\Omega^{†}\right),$$

where

(17) $$\Omega^{2}=\overset{n}{\underset{k=1}{⨂}}R_{k}^{2}=\overset{n-1}{\underset{k=0}{⨂}}R_{k}=I⊗R_{1}⊗\cdots ⊗R_{n-1},$$

since $R_{k}^{2}=R_{k-1}$ and $R_{0}=I$ (one-qubit identity gate), see Equation (13).

As a final step in optimizing our quantum circuit, we want to make the SWAP operations, usually required in a proper (I)QFT to obtain the correct states, unnecessary. Therefore, following the argument in [58], it is useful to introduce the SWAP operation on the *n*-qubit register, which we denote by τ. The SWAP takes qubit *k* to qubit n−1−k and vice versa,

(18) $$\tau :\;|q_{n-1}\ldots q_{0}\rangle \mapsto |q_{0}\ldots q_{n-1}\rangle .$$

This operation is unitary, $\tau^{-1}=\tau^{†}$, and involutory, $\tau^{-1}=\tau$. A proper (I)QFT on *n* qubits requires a SWAP on the *n*-qubit register at the end (beginning) of the circuit, i.e.,

(19) $$\begin{array}{c} F=\tau \tilde{F},\;\mathrm{and}\;F^{†}={\tilde{F}}^{†}\tau , \end{array}$$

where ${\tilde{F}}^{\left(†\right)}$ denotes the (I)QFT without the SWAP. Similarly, we introduce

(20) $$\tilde{\Omega }=\tau \Omega \tau =R_{n}⊗R_{n-1}⊗\cdots ⊗R_{1},$$

see Equation (12). Using Equations (19)–(20) and recalling that $\tau^{-1}=\tau^{†}=\tau$ acts only on the position register (not on the coin), we observe that

$$\begin{array}{cc} \left(I_{c}⊗F^{†}\right) & =\left(I_{c}⊗{\tilde{F}}^{†}\right)\left(I_{c}⊗\tau \right),\\ \left(I_{c}⊗F\right) & =\left(I_{c}⊗\tau \right)\left(I_{c}⊗\tilde{F}\right),\\ \Sigma & =\left(I_{c}⊗\tau \right)(|0_{c}\rangle \langle 0_{c}|⊗I_{p}+|1_{c}\rangle \langle 1_{c}|⊗{\tilde{\Omega }}^{2})\left(I_{c}⊗{\tilde{\Omega }}^{†}\right)\left(I_{c}⊗\tau \right),\\ \left(C⊗I_{p}\right) & =\left(I_{c}⊗\tau \right)\left(C⊗I_{p}\right)\left(I_{c}⊗\tau \right), \end{array}$$

according to which we can rewrite Equation (15) as

(21) $$\begin{array}{cc} U^{t} & =\left(I_{c}⊗{\tilde{F}}^{†}\right){\left[(|0_{c}\rangle \langle 0_{c}|⊗I_{p}+|1_{c}\rangle \langle 1_{c}|⊗{\tilde{\Omega }}^{2})\left(C⊗{\tilde{\Omega }}^{†}\right)\right]}^{t}\left(I_{c}⊗\tilde{F}\right). \end{array}$$

**Figure 4:**
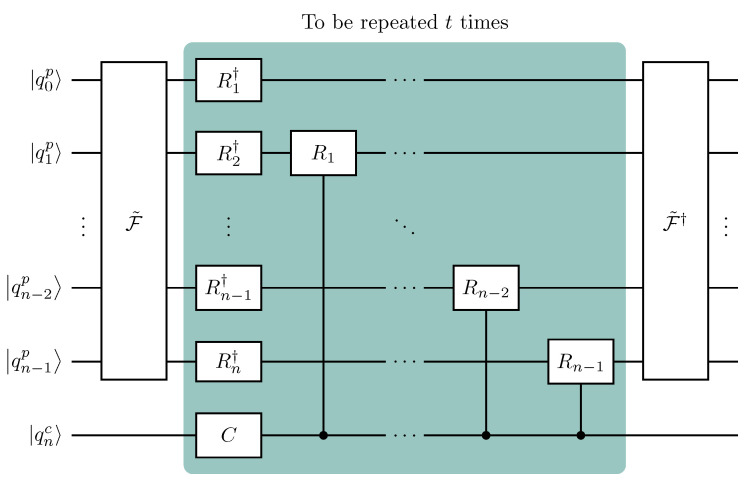
Quantum circuit implementing one time step of the DTQW on the $2^{n}$-cycle proposed in the present work. The quantum Fourier transform, $\tilde{F}$, and its inverse, ${\tilde{F}}^{†}$, do *not* include the SWAP gates. To implement *t* time steps of the DTQW, we have to concatenate the above circuit *t* times. In doing so, since the QFT is unitary, ${\tilde{F}}^{†}\tilde{F}=I$, we are left with only one QFT at the beginning, the central block (shaded) repeated *t* times, and one IQFT at the end. This simplification cannot occur in the quantum circuit in Figure 3 due to the CNOT and the coin gates. For an initially localized walker, $|\psi_{0}\rangle =|\phi_{c}\rangle ⊗|0_{p}\rangle$, the initial QFT is conveniently replaced by a layer of Hadamard gates (see Appendix B).

In conclusion, the quantum circuit implementing *t* steps of a DTQW, excluding the initial state preparation, is shown in Figure 4 and does not need the SWAP in the (I)QFT. Size and depth of a quantum circuit implementing a DTQW can be further reduced by choosing a proper encoding of the position space and designing initial-state-dependent circuits [60]. We point out that the design of our quantum circuit is independent of the initial state, but it can be further optimized for an initially localized walker, the usual initial condition, by replacing the initial QFT with a layer of Hadamard gates (Appendix B).

### 3.2. Comparison with Other Existing Schemes

In this section, we estimate the size of the proposed quantum circuit (Figure 4) in terms of depth D and number of one- and two-qubit gates, $N^{\left(1\right)}$ and $N^{\left(2\right)}$, respectively, and compare it with those of other existing schemes, following the preliminary analysis provided in [58]. We compare our scheme with the following ones: (i) The ID scheme [48], which is based on the increment and decrement gates (Figure 2) that require generalized CNOT gates. The latter can be implemented in different ways, so, as an example, we consider their implementation (i.a) via linear-depth quantum circuit [61] or (i.b) via ancilla qubits [62]. In passing, we also mention a possible implementation via rotations [56]. In the following analysis, we consider the ID scheme implemented as in Figure 2d, with the increment gate only. (ii) The QFT scheme [58], which is based on the increment gate diagonalized by the QFT (Figure 3).

**Figure 5:**
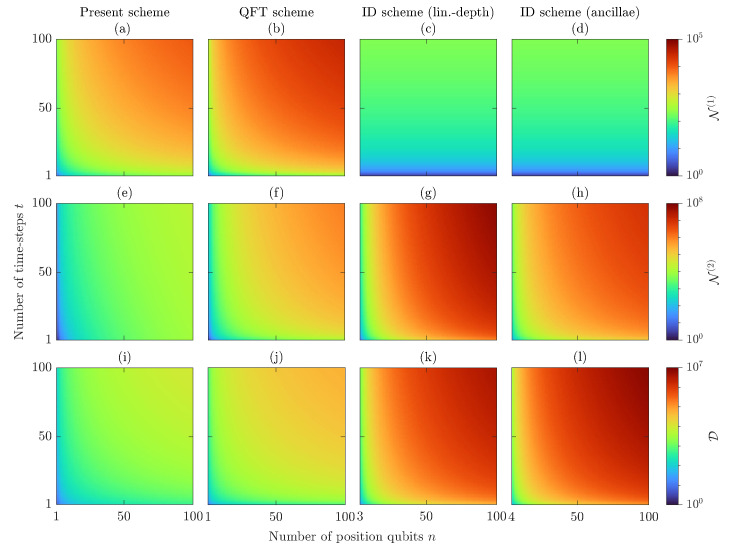
Metrics in Table 1 as a function of the number of position qubits, *n*, and the number of time steps, *t*, of a DTQW on the $2^{n}$-cycle. Each column corresponds to a different scheme: (**a**,**e**,**i**) Present, (**b**,**f**,**j**) QFT, (**c**,**g**,**k**) ID (lin.-depth), and (**d**,**h**,**l**) ID (ancillae). Each row corresponds to a different metric: (**a**–**d**) Number of one-qubit gates $N^{\left(1\right)}$, (**e**–**h**) Number of two-qubit gates $N^{\left(2\right)}$, and (**i**–**l**) circuit depth D.

This discussion is neither supposed to be exhaustive, e.g., there are several ways to implement the generalized CNOT gates in the ID scheme, nor supposed to provide optimal and universal metrics, as the latter are ultimately quantum-device-dependent, e.g., it suffices to think of the process of transpilation, which rewrites and/or optimizes a given circuit according to the topology of the quantum device considered. Still, our estimate is useful to show how our scheme scales better than others when including the number of implemented time steps in the analysis, in particular if compared to the QFT scheme (Figure 3), which is, to the best of our knowledge, the most efficient state-of-the-art implementation of DTQW on $2^{n}$-cycles. Results of the circuit size in the different schemes are summarized in Table 1 and shown in Figure 5. Details of the computation are deferred to Appendix C.

Considering both the number *n* of position qubits and the number *t* of time steps, the ID scheme is the most resource-demanding among those examined: (i.a) If generalized CNOT gates are implemented via linear-depth quantum circuits, then the circuit’s depth increases as $D=O(4tn^{2})$ and the results quickly degrade due to the large number of two-qubit gates, $N^{\left(2\right)}=O\left(2tn^{3}/3\right)$. (i.b) If generalized CNOT gates are implemented via ancilla qubits, then the circuit’s depth is of the same order, $D=O(8tn^{2})$, but the number of two-qubit gates is reduced by an order, $N^{\left(2\right)}=O\left(10tn^{2}\right)$, at the cost of requiring extra qubits, $N^{\left(a\right)}=O\left(n\right)$. Both the ID approaches require $N^{\left(1\right)}=2t$. (ii) A remarkable improvement is obtained by the QFT scheme, which, with no need of ancilla qubits, has D=O(6tn) and $N^{\left(2\right)}=O\left(tn^{2}\right)$, at the cost of increasing the number of one-qubit gates, $N^{\left(1\right)}=O\left(3tn\right)$. Our scheme refines such metrics by making the cost of the (I)QFT independent of the number of time steps: in the long time limit, t≫n, we have $D=N^{\left(1\right)}=N^{\left(2\right)}=O\left(tn\right)$, to which is added a fixed, *t*-independent but *n*-dependent, cost D=O(4n), $N^{\left(2\right)}=O\left(n^{2}\right)$, and $N^{\left(1\right)}=O\left(2n\right)$. To ease the comparison among the schemes, the metrics of Table 1 are shown in Figure 5, making it clear that our scheme outperforms the others in the number of two-qubit gates, which take the longest time to execute and are the noisiest in quantum computers. Furthermore, we observe that the circuit depth is mainly determined by the number of two-qubit gates.

In conclusion, both the QFT scheme and ours outperform the ID scheme at any time. Although these metrics are comparable in the few-time-steps regime, our scheme outperforms the QFT scheme when a large number of time steps is implemented.

## 4. Results and Discussion

**Figure 6:**
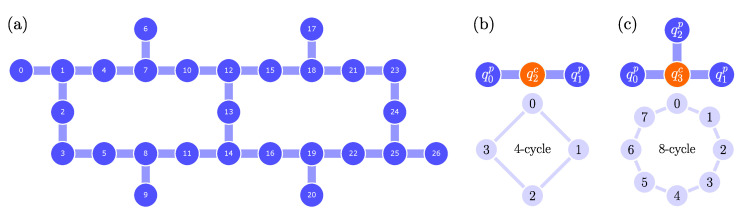
(**a**) Qubit connectivity map of ibm_cairo. (**b**,**c**) Optimal mapping of the coin–position state onto multiqubit state $|q_{n}^{c}q_{n-1}^{p}\ldots q_{0}^{p}\rangle$ for the cycle with (**b**) N=4 and (**c**) N=8 vertices (n=2,3 position qubits, respectively). No SWAP operations between position and coin qubits are required by the controlled-$R_{k}$ gates in Figure 4, the coin qubit (orange) being already adjacent to all position qubits (blue).

We test the DTQW circuit introduced in Section 3.1 (see Figure 4) on the IBM quantum computer ibm_cairo v.1.3.5, a 27-qubit Falcon r5.11 processor whose qubit connectivity map is shown in Figure 6a. The IBM Quantum system ibm_cairo is remotely accessed via the IBM cloud computing services [63] and experiments are run within the Qiskit framework [64]. In the following, we introduce the DTQW considered for the test and the quantities of interest, then we point out some solutions to improve the circuit, and finally we present and discuss the results.

### 4.1. Hadamard DTQW

A common choice for the coin operator is the Hadamard coin

(22) $$C=\frac{1}{\sqrt{2}}\left(\begin{array}{cc} 1 & 1\\ 1 & -1 \end{array}\right).$$

As for the initial state, we assume the walker to be initially localized in $|0_{p}\rangle$ and the coin to be in a given superposition

(23) $$|\psi_{0}\rangle =\left[\cos\left(\frac{\pi }{12}\right)|0_{c}\rangle +i\sin\left(\frac{\pi }{12}\right)|1_{c}\rangle \right]|0_{p}\rangle .$$

The Hadamard DTQW for this initial state has the following properties: (i) The dynamics is periodic of period $T_{\mathrm{dyn}}=8$ ($T_{\mathrm{dyn}}=24$) on the 4-cycle (8-cycle) [65]. (ii) Maximally entangled single-particle states—entanglement between position and coin—are generated after one step of the walk and then recurrently with period $T_{\mathrm{MESPS}}=4$ ($T_{\mathrm{MESPS}}=12$) on the 4-cycle (8-cycle) [59]. These properties are therefore suitable for thoroughly testing the quality of the designed quantum circuit, i.e., to assess to what extent the actual implementation can reproduce these ideal features. We point out that for a Hadamard DTQW on the 4- and 8-cycles, any initial state (6) with ϕ=π/2 will generate a dynamics with the two above-mentioned features for any value of θ [59]. We arbitrarily set θ=π/6 to initialize the coin in a nontrivial superposition of states, see Equation (23). For later convenience, we anticipate that the periodic dynamics of the DTQW on the 4- and 8-cycles will sustain the periodic occurrence of localized states when the initial state is localized in position space. However, dynamics and occurrence of localized states can have different periods. In the 4-cycle, the initial state localized in $|0_{p}\rangle$ is perfectly transferred to $|2_{p}\rangle$ after four time steps; hence, localized states occur with period $T_{\mathrm{dyn}}/2=4$, in contrast with the period $T_{\mathrm{dyn}}=8$ of the dynamics. Instead, in the 8-cycle, localized states occur only as a result of the periodic dynamics ($T_{\mathrm{dyn}}=24$) [59]. Implementing the cycle with N=4 and N=8 vertices requires n=2 and n=3 position qubits, respectively.

### 4.2. Figures of Merit

*Probability distribution*: In most DTQW problems, we are interested in the probability distribution of the walker’s position. Our purpose is to compare the ideal probability distribution with the experimental ones, the latter obtained in a noisy simulation and in an actual implementation on the quantum hardware ibm_cairo. To compare two discrete probability distributions, $P={\left\{p_{k}\right\}}_{k}$ and $Q={\left\{q_{k}\right\}}_{k}$, we adopt the Hellinger fidelity $H\left(P,Q\right)={\left[1-h^{2}\left(P,Q\right)\right]}^{2}$, where the Hellinger distance h(P,Q) [66] between *P* and *Q* is defined by

(24) $$h\left(P,Q\right)=\frac{1}{\sqrt{2}}\sqrt{\sum_{k}{\left(\sqrt{p_{k}}-\sqrt{q_{k}}\right)}^{2}}.$$

The Hellinger distance is symmetric, h(P,Q)=h(Q,P), and bounded 0≤h(P,Q)≤1, where the value h=0 means that the two distributions are equal (fidelity H=1).

*Entanglement*: Usually, in a DTQW, entanglement between walker and coin occurs. This can be understood as hybrid entanglement, also referred to as single-particle entanglement because it is established between different degrees of freedom of the same quantum system, here position and coin, which is an internal degree of freedom, e.g., spin [67]. Bipartite entanglement can be probed by means of entanglement entropies. In this case, we probe the second-order Rényi entanglement entropy [68] of the reduced density matrix for the two parts (coin and position) via randomized measurements [69]. Estimating this quantity requires significantly fewer measurements than performing quantum state tomography: for an *n*-qubit state, $O(2^{an})$ with a<2 (the coefficient *a* depends on the nature of the considered state, see Supplementary Materials for [69]) compared to $2^{2n}-1$ [70]. In this regard, the advantage of this approach becomes remarkable when a large number of qubits is involved—as expected to be in future applications—due to the current costliness of tomography. The second-order Rényi entropy for a part *A* of the total bipartite system described by $\rho_{AB}$ is defined as

(25) $$S^{\left(2\right)}\left(\rho_{A}\right)=-\log_{2}\left(\rho_{A}^{2}\right),$$

where $\rho_{A}{=}_{B}\left(\rho_{AB}\right)$ is the reduced density matrix for part *A*. If the second-order Rényi entropy of the part is greater than that of the total system, $S^{\left(2\right)}\left(\rho_{A}\right)>S^{\left(2\right)}\left(\rho_{AB}\right)$, then bipartite entanglement exists between the two parts (for separable states $S^{\left(2\right)}\left(\rho_{A}\right)\leq S^{\left(2\right)}\left(\rho_{AB}\right)$ and $S^{\left(2\right)}\left(\rho_{B}\right)\leq S^{\left(2\right)}\left(\rho_{AB}\right)$ [68]). If, in addition, the overall state $\rho_{AB}$ is pure, then the second-order Rényi entropy is directly a measure of bipartite entanglement and $S^{\left(2\right)}\left(\rho_{A}\right)=S^{\left(2\right)}\left(\rho_{B}\right)$ (the reduced density matrices of a pure bipartite state have the same nonzero eigenvalues, due to the Schmidt decomposition). The second-order Rényi entropy is maximum for the maximally mixed state, $\max_{\rho_{A}}S^{\left(2\right)}\left(\rho_{A}\right)=\log_{2}d_{A}$, with $d_{A}$ the dimension of $\rho_{A}$. Furthermore, $S^{\left(2\right)}\left(\rho_{AB}\right)$ is indicative of the overall purity of the system, because it is null for pure quantum states.

### 4.3. Circuit Optimization

Each time step of the DTQW requires the coin qubit to interact with each position qubit (see controlled-$R_{k}$ gates in Figure 4). The quantum hardware may have limited connectivity, therefore SWAP operations are needed to make two qubits interact whenever the latter are not physically adjacent. A wise circuital implementation must account for the connectivity of the quantum hardware considered. We can limit the number of SWAPs by making the coin qubit as “shared” as possible, compatibly with the typical sparse connectivity of superconducting quantum computers. The qubit topology of ibm_cairo (Figure 6a) makes it possible to implement the circuit for a DTQW on the 4- and 8-cycles without SWAPs by mapping coin and position qubits as in Figure 6b and c, respectively. Given the optimal mapping that is compatible with the given qubit connectivity, we consider the set of qubits having the lowest error rates averaged over different calibrations. In addition, the initial state (23) being localized in position space, we replace the initial QFT with a layer of Hadamard gates (Appendix B).

### 4.4. Analysis of the DTQW on the 4- and 8-Cycles

**Figure 7:**
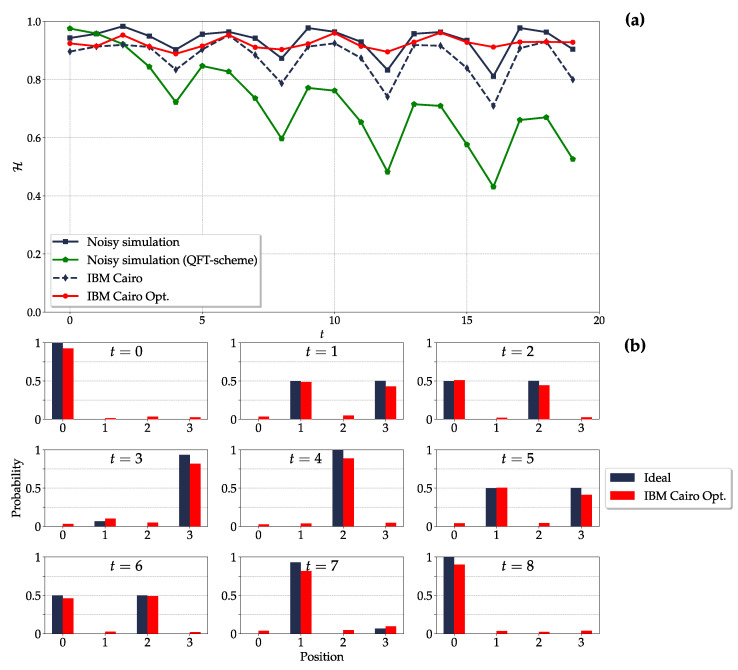
Hadamard DTQW on the (N=4)-cycle with initial state as in Equation (23). (**a**) Hellinger fidelity between the ideal and the experimental probability distributions of walker’s position as a function of time step *t*. The experimental distributions include noisy simulation and implementations on the actual quantum hardware with optimization_level=1 (IBM Cairo) and optimization_level=3 (IBM Cairo Opt.) in transpilation. Results for the noisy simulation of the DTQW circuit in the QFT scheme [58] are reported for comparison. (**b**) Ideal and experimental (IBM Cairo Opt.) probability distributions of walker’s position for time steps t=0,…,8. Results for both simulations and quantum hardware implementation are obtained for $10^{5}$ shots and by encoding the position state in qubits 3 and 8 and the coin state in qubit 5 of ibm_cairo, see Figure 6a,b.

*Probability distribution (4-cycle)*: Performing a noisy simulation of the quantum circuit is the preliminary step before the actual implementation on the quantum hardware. Figure 7a shows the Hellinger fidelities of the walker’s position distribution on the 4-cycle. The Hellinger fidelity for the noisy simulation of our circuit is above 80% for all the 19 time steps implemented, while the results for the noisy simulation of the circuit in the QFT scheme [58] degrade below the 80% after a few steps. The previous analysis on the circuit size proved the advantage of our circuit in the long time limit, but these results suggest that our circuit outperforms the QFT scheme circuit already in the few-time-steps regime. For the actual implementation on ibm_cairo, we consider two levels of optimization in the transpilation: (i) optimization_level=1 (default value), which transpiles the circuit into the native gates of the hardware and performs a light optimization (blue dashed line, curve “IBM Cairo”), and (ii) optimization_level=3, which performs the heaviest optimization (red solid line, curve “IBM Cairo Opt.”).

The Hellinger fidelity for the default implementation of our circuit (optimization_level=1) shows a moderate discrepancy with respect to the noisy simulation but closely follows the trend of the latter. The heavily optimized implementation of our circuit (optimization_level=3) provides better results and also partially mitigates the local minima of the noisy simulation. However, we point out that the circuit transpiled with the highest optimization level turns out to have a depth which is basically independent of the number of time steps implemented (see Appendix D). This explains the long-lasting optimality of the results, H≳90% up to t=19 (last time step implemented). However, this optimal transpiled circuit is obtained only for n=2 position qubits (4-cycle); for n=3 (8-cycle), we obtain a circuit whose depth increases with the number of time steps. The Hellinger fidelity at t=0 is lower than 1 because we still implement the whole circuit with t=0, i.e., we do not just implement the initial state. Furthermore, the Hellinger fidelity is characterized by periodic local minima, with period 4 being half of the period of the dynamics. These minima occur when the walker is ideally localized in position, a probability distribution so peaked (delta) that it can hardly be obtained as the result of an actual, noisy implementation (see Figure 7b). The frame t=8 in Figure 7b shows that the walker has returned to the initial position, as expected for the periodic dynamics.

**Figure 8:**
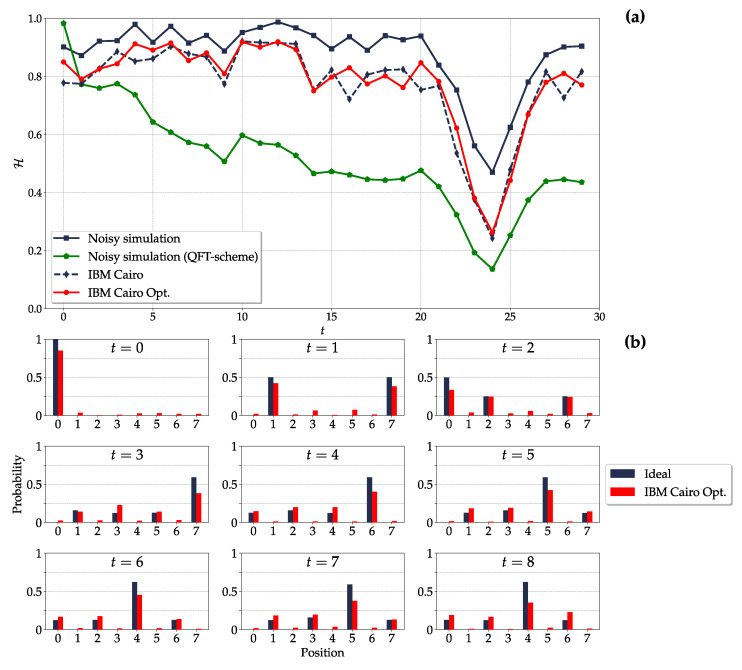
The same as in Figure 7 but for N=8. Results for both simulations and quantum hardware implementation are obtained for $10^{5}$ shots and by encoding the position state in qubits 10, 13, and 15 and the coin state in qubit 12 of ibm_cairo, see Figure 6a,c.

*Probability distribution (8-cycle)*: We obtain qualitatively analogous results also for the DTQW on the 8-cycle (Figure 8). However, as anticipated in the previous paragraph, in this case, the optimization in the transpilation is not as effective as for the 4-cycle. The Hellinger fidelities with optimization_level=1 and 3 are thus consistent with each other. Unlike the DTQW on the 4-cycle, given an initial localized state, localized states at later times occur only as a result of the periodic dynamics ($T_{\mathrm{dyn}}=24$), hence the minimum of the Hellinger fidelity at t=24.

**Figure 9:**
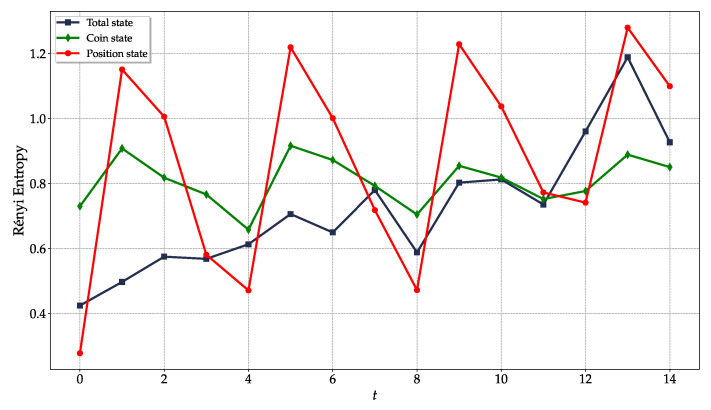
Recurrent generation of maximally entangled single-particle states for a Hadamard DTQW on the (N=4)-cycle with initial state as in Equation (23) investigated by means of the second-order Rényi entropy as a function of time steps *t*. Bipartite entanglement between the two parts (coin and position degrees of freedom) exists if the second-order Rényi entropy of a part is larger than that of the total system. Results of entropies are obtained via 300 randomized measurements [69] and $10^{5}$ shots for each step of the DTQW, with optimization_level=1 in transpilation. Position state is encoded in qubits 3 and 8 and the coin state in qubit 5 of ibm_cairo, see Figure 6a,b.

*Entanglement (4-cycle)*: Probing the second-order Rényi entanglement entropy via randomized measurements [69] for many time steps of the DTQW and for increasing size of the cycle is computationally demanding. Therefore, to discuss the recurrent generation of maximally entangled single-particle states, we focus only on the DTQW on the 4-cycle (Figure 9). First, we review the ideal scenario. Given an initial pure state and a unitary evolution, the second-order Rényi entropy of coin state, $\rho_{c}$, and position state, $\rho_{p}$, are equal, $S^{\left(2\right)}\left(\rho_{c}\right)=S^{\left(2\right)}\left(\rho_{p}\right)$, and that of the total system, $\rho_{cp}$, is null (pure state) at any time *t*. Furthermore, we have $S_{\max}^{\left(2\right)}\left(\rho_{c}\right)=S_{\max}^{\left(2\right)}\left(\rho_{p}\right)=\log_{2}\left(\min\left\{d_{c},d_{p}\right\}\right)=1$, the two-dimensional coin being the part with the lowest dimension. The initial state $|\psi_{0}\rangle$ in Equation (23) is pure and separable (no entanglement), as is the state at t=4

(26) $$|\psi_{4}\rangle =\left[\cos\left(\frac{\pi }{12}\right)|0_{c}\rangle +i\sin\left(\frac{\pi }{12}\right)|1_{c}\rangle \right]|2_{p}\rangle ,$$

which is $|\psi_{0}\rangle$ perfectly transferred in position space from $|0_{p}\rangle$ to $|2_{p}\rangle$. The dynamics has period $T_{\mathrm{dyn}}=8$, thus $S^{\left(2\right)}\left(\rho_{\alpha }\right)=0$ at t=4k with k=0,1,2,… and α=c,p, denoting the absence of entanglement when periodic separable states occur. In addition, maximally entangled single-particle states are generated at t=1 and t=5

(27) $$\begin{array}{cc} |\psi_{1}\rangle & =\frac{1}{\sqrt{2}}\left[e^{i\pi /12}|0_{c}\rangle |3_{p}\rangle +e^{-i\pi /12}|1_{c}\rangle |1_{p}\rangle \right], \end{array}$$

(28) $$\begin{array}{cc} |\psi_{5}\rangle & =\frac{1}{\sqrt{2}}\left[e^{i\pi /12}|0_{c}\rangle |1_{p}\rangle +e^{-i\pi /12}|1_{c}\rangle |3_{p}\rangle \right], \end{array}$$

and are later supported by the periodic dynamics, thus $S^{\left(2\right)}\left(\rho_{\alpha }\right)=1$ at t=1+4k with k=0,1,2,… and α=c,p. For the remaining time steps, t=2+4k and t=3+4k, partially entangled states are expected (see Supplementary Materials of [59]).

Experimentally (Figure 9), the second-order Rényi entropy of the total bipartite system is nonzero even at t=0 and increases with time, denoting a further degradation of the purity of the state. Since the latter is not pure, in general we expect $0<S^{\left(2\right)}\left(\rho_{cp}\right)\leq 3$ (the upper bound refers to the maximally mixed three-qubit state), $S^{\left(2\right)}\left(\rho_{c}\right)\neq S^{\left(2\right)}\left(\rho_{p}\right)$ and different maximum values, $S^{\left(2\right)}\left(\rho_{c}\right)\leq \log_{2}d_{c}=1$ and $S^{\left(2\right)}\left(\rho_{p}\right)\leq \log_{2}d_{p}=2$ (4-cycle). Whenever separable states are expected (t=4k), we observe local minima of $S^{\left(2\right)}\left(\rho_{\alpha }\right)$, but either part has $S^{\left(2\right)}\left(\rho_{\alpha }\right)>S^{\left(2\right)}\left(\rho_{cp}\right)$, thus denoting the presence of residual entanglement. Whenever maximally entangled single-particle states are expected (t=1+4k), we observe local maxima of $S^{\left(2\right)}\left(\rho_{\alpha }\right)$, with α=c,p, both of which are greater than $S^{\left(2\right)}\left(\rho_{cp}\right)$ up to t=9. For the remaining time steps, we observe that at least either part has $S^{\left(2\right)}\left(\rho_{\alpha }\right)≳S^{\left(2\right)}\left(\rho_{cp}\right)$, consistent with the expected presence of partial entanglement.

To summarize, the actual multiqubit state implemented on the quantum hardware and then processed by the quantum circuit is generally mixed and characterized by residual entanglement, but the local minima and maxima of $S^{\left(2\right)}\left(\rho_{c}\right)$ and $S^{\left(2\right)}\left(\rho_{p}\right)$ are perfectly consistent with the expected periodic separable and maximally entangled single-particle states, respectively, up to t=9. Results suggest that the observed local maxima will preserve the expected periodicity for further steps t>9, but eventually $S^{\left(2\right)}\left(\rho_{cp}\right)\geq S^{\left(2\right)}\left(\rho_{\alpha }\right)$ due to the degradation of the purity of the total state. Entanglement is a distinct quantum signature and, in this example, we have clear evidence of generation up to t=9. This does not necessarily imply that quantum features, including entanglement, in the realization of the DTQW are lost thereafter. Indeed, for the remaining time steps investigated, 9<t≤14, we still observe $S^{\left(2\right)}\left(\rho_{p}\right)≳S^{\left(2\right)}\left(\rho_{cp}\right)$—presence of entanglement—whenever states with partial or maximum entanglement are expected. Therefore, quantum features are present up to the last time step considered, t=14.

## 5. Conclusions

We proposed an efficient quantum circuit for the DTQW on the $2^{n}$-cycle. Our scheme, using only one QFT and one IQFT, significantly improves the most efficient state-of-the-art implementation [58] (referred to as QFT scheme in the following) which uses one QFT and one IQFT at *each* time step. As a result, our circuit requires only $O(n^{2}+nt)$ two-qubit gates, compared to the $O(n^{2}t)$ of the QFT scheme. The improvement in this gate count is even more significant for long times, passing, for t≫n, from $O(n^{2}t)$ in the QFT scheme to O(nt) in ours. Therefore, since two-qubit gates take the longest time to execute and are the noisiest, our quantum circuit is computationally less demanding and paves the way for reliable implementation in noisy–intermediate-scale quantum devices [71,72,73].

In this regard, we tested the proposed quantum circuit on actual quantum hardware, ibm_cairo, considering a Hadamard DTQW on the 4- and 8-cycles. Both are characterized by periodic dynamics [65] and by recurrent generation of maximally entangled single-particle states [59]. We claim two main results. First, even in the short time regime, the present quantum circuit outperforms the current state-of-the-art DTQW circuits, whose results are degraded after only a few steps [56,58]. Despite the moderate discrepancy, our implementation on actual quantum hardware provides results that closely follow those from the noisy simulation. In particular, the Hellinger fidelity between the ideal probability distribution of the walker’s position and the experimental one is above 90% for all the t=19 time steps we implemented in the 4-cycle and above 80% up to t=13 time steps in the 8-cycle. Second, for the DTQW on the 4-cycle, we provide experimental evidence of the recurrent generation of nearly maximally entangled single-particle states up to t=9 time steps. The expected maximum entanglement is not achieved because the ideally pure state of the bipartite system is actually implemented on the quantum hardware as a mixed multiqubit state, and its purity degrades over time.

The implementation of our circuit on actual quantum computers may benefit from the following. The circuit strongly relies on controlled $R_{k}$-gates (phase-shift gate), and the latter can be efficiently implemented using a single ancillary qubit [74]. Moreover, as the position space increases, the sparse connectivity of a superconducting quantum computer results in large experimental overheads of SWAP gates, which becomes unavoidable, e.g., to make the coin qubit interact with each position qubit. In this regard, a virtual two-qubit gate can be employed to suppress errors due to the additional SWAP gates [75]. Alternatively, an implementation on a quantum hardware architecture with full connectivity [76,77,78] may be more advantageous.

Possible applications of our scheme include the circuital implementation of direct communication protocols [35,36] and quantum key distribution protocols [38] based on DTQW on the cycle. We point out that the proposed circuit does not impose constraints on the coin operator (one-qubit gate), which in principle can be changed at each time step. Parrondo’s paradox arises when losing strategies are combined to obtain a winning one and it cuts across various research areas. This counterintuitive phenomenon can be observed in DTQW on the line or cycle when two or more coin operators are applied in a deterministic sequence [79,80]. Therefore, we expect that our quantum circuit may also be of interest to quantum game theory [81].

Circuit implementations of DTQW on a cycle of arbitrary *N* have been addressed in [51,58], while our proposal is limited to DTQW on the *N*-cycle with $N=2^{n}$. We point out, however, that any circulant matrix is diagonalized by the QFT, and this has been already exploited to efficiently implement CTQWs on circulant graphs [82]. Similarly, our implementation for DTQWs can be generalized to circulant graphs—graphs whose adjacency matrix is circulant—which, being *d*-regular (each vertex has degree *d*), will require a *d*-dimensional coin [83,84]. A generalization of our approach to more complex structures is therefore desirable and potentially of larger interest, e.g., for algorithmic purposes.

## Figures and Tables

**Table 1:** Metrics of the quantum circuit implementing *t* time steps of a DTQW on the $2^{n}$-cycle for different schemes: $N^{\left(1\right)}$ and $N^{\left(2\right)}$ denote the number of one- and two-qubit gates, respectively, D the depth of the circuit, and $N^{\left(a\right)}$ the number of ancilla qubits. The number *n* refers to the number of qubits encoding the walker’s position. See also Figure 5.

Scheme	Figure	One-Qubit $N^{\left(1\right)}$	Two-Qubit $N^{\left(2\right)}$	Depth D	Ancillae $N^{\left(a\right)}$
Present work	4	t(n+1)+2n	t(n−1)+n(n−1)	tn+2(2n−1)	0
QFT [58]	3	t(3n+1)	tn(n+1)	t(6n)	0
ID * [48] (lin.-depth q.c. [61], n≥3)	2d	2t	$t\frac{1}{3}\left(2n^{3}-6n^{2}+13n-3\right)$	$t(4n^{2}-14n+19)$	0
ID * [48] (ancillae [62], n≥4)	2d	2t	$t(10n^{2}-48n+66)$	$t(8n^{2}-38n+55)$	n−3

* These schemes differ in how the generalized CNOT gates in the increment gate are realized (see Figure 2b).

**Figure A1:**
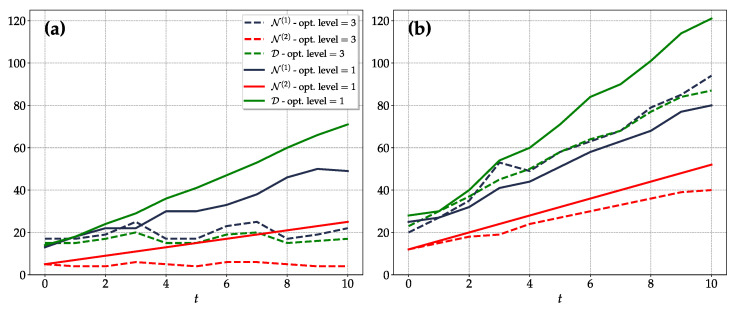
Gate count in the quantum circuits proposed in the present work for the Hadamard DTQW on the (**a**) 4-cycle and (**b**) 8-cycle (Section 4.1): depth D and number of one- and two-qubit gates, $N^{\left(1\right)}$ and $N^{\left(2\right)}$, respectively, as a function of time steps *t* with optimization_level=1,3 in transpilation (ibm_cairo). The quantum circuits for t=1 are shown in Figure A2 (4-cycle) and Figure A3 (8-cycle).

**Figure A2:**
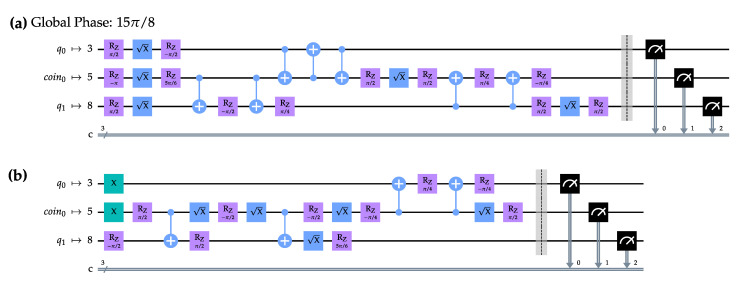
Quantum circuit proposed in the present work (Figure 4) for one step of the Hadamard DTQW (Section 4.1) on the 4-cycle transpiled in ibm_cairo with (**a**) optimization_level=1 and (**b**) optimization_level=3. Each qubit is labelled by the corresponding index in the qubit connectivity map in Figure 6a.

**Figure A3:**
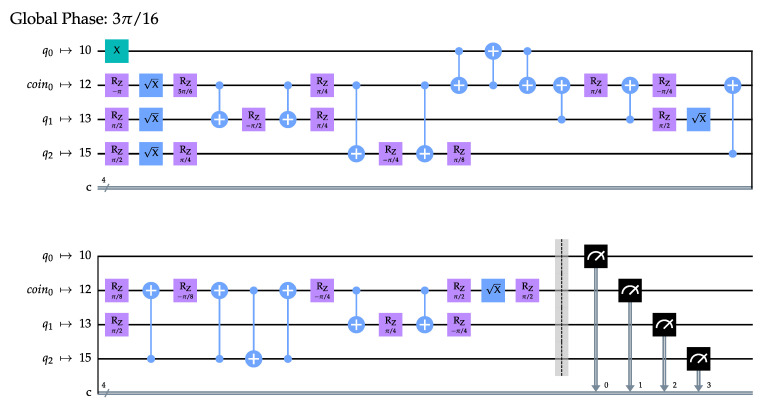
The same as in Figure A2, but for the 8-cycle with optimization_level=1.

## Data Availability

The dataset generated and analyzed in the current study is available from the corresponding author upon reasonable request.

## Abbreviations

    The following abbreviations are used in this manuscript:

CTQW	Continuous-time quantum walk
DTQW	Discrete-time quantum walk
(I)QFT	(Inverse) quantum Fourier transform

## Appendix A. Eigenvalues and Eigenvectors of Circulant Matrices

Let *C* be an N×N circulant matrix [85]

(A1) $$C=\left(\begin{array}{ccccc} c_{0} & c_{1} & \ldots & c_{N-2} & c_{N-1}\\ c_{N-1} & c_{0} & c_{1} & \; & c_{N-2}\\ \vdots & c_{N-1} & c_{0} & \ddots & \vdots \\ c_{2} & & \ddots & \ddots & c_{1}\\ c_{1} & c_{2} & \ldots & c_{N-1} & c_{0} \end{array}\right),$$

where each row is a cyclic shift of the row above it. The structure can also be characterized by noting that

(A2) $$C_{k,j}=c_{(j-k)\;\mathrm{mod}\;N}.$$

The eigenvalues $\lambda_{m}$ and eigenvectors $y^{\left(m\right)}$ (column vector) of *C*, solutions of $Cy^{\left(m\right)}=\lambda_{m}y^{\left(m\right)}$, are

(A3) $$\begin{array}{cc} \lambda_{m} & =\sum_{k=0}^{N-1}c_{k}\omega_{N}^{-mk}, \end{array}$$

(A4) $$\begin{array}{cc} y^{\left(m\right)} & =\frac{1}{\sqrt{N}}{\left(1,\omega_{N}^{-1},\omega_{N}^{-2},\ldots ,\omega_{N}^{-(N-1)}\right)}^{⊺}, \end{array}$$

with $\omega_{N}=\exp\left(2\pi i/N\right)$ and the symbol ^⊺^ denoting the transpose without complex conjugation. Letting $\Lambda =\mathrm{diag}({\left\{\lambda_{m}\right\}}_{m})$, we have

(A5) $$C=U\Lambda U^{†},\;i.e.,\;\Lambda =U^{†}CU,$$

where *U* is the unitary matrix

(A6) $$U=\left(y^{\left(0\right)}\left|y^{\left(1\right)}\right|\cdots \left|y^{\left(N-1\right)}\right.\right)$$

whose elements read $U_{j,k}=\omega_{N}^{-jk}/\sqrt{N}$, with j,k=0,1,…,N−1. Since they have the same definition, in $U^{†}$ and *U* we recognize, respectively, the quantum Fourier transform, F, and its inverse, $F^{†}$. Therefore, in our framework, diagonalizing a circulant matrix *C* satisfies

(A7) $$C=F^{†}\Lambda F,\;i.e.,\;\Lambda =FCF^{†}.$$

As a case study, we consider the eigenproblem for the circulant matrices $P_{0}$ and $P_{1}$ in Equation (5). According to the notation introduced above for circulant matrices (A1) and their eigenvalues (A3), the matrix $P_{0}$ is defined by the elements $c_{m}=\delta_{m,1}$, its eigenvalues are $\lambda_{m}=\omega_{N}^{-m}$, and so its diagonal form is the matrix $\Omega^{†}$ with Ω defined in Equation (12). The matrix $P_{1}$ is defined by the elements $c_{m}=\delta_{m,N-1}$, its eigenvalues are $\lambda_{m}=\omega_{N}^{-(N-1)m}=\omega_{N}^{m}$, since $\omega_{N}^{-Nm}=\exp\left(-2\pi im\right)=1$, and so its diagonal form is the matrix Ω.

## Appendix B. Optimization of the Circuit for an Initially Localized Walker

The initial state of a DTQW is typically assumed to be in the factorized form

(A8) $$|\psi_{0}\rangle =|\phi_{c}\rangle |0_{p}\rangle .$$

The coin is in a generic state $|\phi_{c}\rangle \in H_{c}^{\left(2\right)}$, which can be realized by a proper unitary, $|\phi_{c}\rangle =U_{c}^{\left(0\right)}|0_{c}\rangle$, starting from the default qubit state initialized to |0〉. Without loss of generality, the walker is localized in the 0-th vertex. Indeed, any DTQW with an initially localized walker can be mapped onto a DTQW with a walker initialized in $|0_{p}\rangle$ because of the equivalence, under cyclic permutation, of all the vertices in a cycle. According to Equation (15), the first matrix acting on the initial state performs a QFT on the walker’s position register. If the walker was in a generic position state, then the whole QFT circuit would be required (see Figure 4). However, given the initial state (A8) and Equation (10), we observe that

(A9) $$\mathrm{QFT}:\;|0\rangle \mapsto \frac{1}{\sqrt{N}}\sum_{k=0}^{N-1}|k\rangle =\overset{n-1}{\underset{m=0}{⨂}}H_{m}{|0\rangle }^{⊗n},$$

where $H_{m}$ is the Hadamard gate $H=\frac{1}{\sqrt{2}}\left(\begin{array}{cc} 1 & 1\\ 1 & -1 \end{array}\right)$ acting on the *m*-th qubit and $N=2^{n}$. Therefore, assuming the initial state (A8), we simply need a layer of Hadamard gates. This is much more convenient than using the whole QFT circuit, because Hadamard gates are one-qubit gates and can thus be executed in parallel. As a final remark, we point out that the above argument is unaffected from having used (I)QFT without SWAP gates in the circuit in Figure 4, since τ(H⊗…⊗H)τ=H⊗…⊗H with τ defined in Equation (18).

## Appendix C. Analysis of the Size of DTQW Quantum Circuits in Different Schemes

In this appendix, we provide details on the computation of the metrics—depth D, number of one- and two-qubit gates, $N^{\left(1\right)}$ and $N^{\left(2\right)}$, respectively—of the quantum circuits implementing a DTQW on the $2^{n}$-cycle reported in Table 1. A few remarks are as follows:

(i) The ID scheme here considered and the QFT scheme are based on the same circuit design (Figure 2d), in which the coin gate (one-qubit gate) cannot be executed in parallel to the CNOT gates, because it acts on their control qubit. Hence, the coin gate has unit depth and prevents the simplification of CNOT gates when iteratively concatenating the single time-step quantum circuit to obtain the successive steps. Therefore, in Appendix C.1 and Appendix C.2, the analysis focuses on a single time step, because the size of a circuit implementing *t* time steps is *t* times the size of that implementing a single time step.
(ii) Controlled gates having different target qubits but the same control qubit (here, it is the coin) are executed in sequence (not in parallel) in actual quantum computers. This affects the depth of circuits and regards the CNOT gates in the ID and QFT schemes (Appendix C.1 and Appendix C.2, respectively) and the controlled-$R_{k}$ gates in our scheme (Appendix C.3).
(iii) The (I)QFT on an *n*-qubit register (no SWAP) requires *n* one-qubit gates (Hadamard) and n(n−1)/2 two-qubit gates (controlled-$R_{k}$) and has depth 2n−1 [86]. This regards the analysis of the QFT scheme (Appendix C.2) and our scheme (Appendix C.3).

### Appendix C.1. ID Scheme

We refer to the quantum circuit proposed in [48] as the ID scheme (Figure 2a–c), being based on increment and decrement gates. In particular, we assume the circuit to be designed as in Figure 2d, because it was proved to be equivalent but more efficient (lower size and depth) than the standard ID scheme in Figure 2a–c [58]. This design requires only the increment gate (not controlled by the coin qubit) and a sequence of 2n CNOT gates (two-qubit gates, depth 2n; see remark (ii)). In this context, different realizations of the ID scheme stem from different implementations of the generalized CNOT gates in the increment gate. In the following, we will refer to the generalized CNOT on k≥3 qubits as the *k*-Toffoli gate, i.e., a gate where one qubit is flipped conditional on the other $n_{c}=k-1$ (control) qubits being set to 1. A 3-Toffoli gate ($n_{c}=2$) denotes the standard Toffoli gate. The increment gate on the *n*-qubit register (walker’s position, see Figure 2b) requires n−3*k*-Toffoli gates—with k=4,…,n, i.e., $n_{c}=3,\ldots ,n-1$; one Toffoli gate (k=3, i.e., $n_{c}=2$); one CNOT ($n_{c}=1$, two-qubit gate); and one NOT ($n_{c}=0$, one-qubit gate); see Figure 2b.

*Implementation via linear-depth quantum circuit*: A *k*-Toffoli gate, with k≥3, can be implemented using a linear-depth quantum circuit having $N^{\left(1\right)}=0$, $N^{\left(2\right)}=2k^{2}-6k+5$, and D=8k−20 [61]. The increment gate (Figure 2b) has $N_{I}^{\left(1\right)}=1$, $N_{I}^{\left(2\right)}=1+\sum_{k=3}^{n}\left(2k^{2}-6k+5\right)$, and $D_{I}=1+1+\sum_{k=3}^{n}\left(8k-20\right)$. Therefore, the overall quantum circuit implementing the single time step of the DTQW (Figure 2d) has

(A10) $$\begin{array}{cc} \; & N^{\left(1\right)}=1+N_{I}^{\left(1\right)}=2, \end{array}$$

(A11) $$\begin{array}{cc} \; & N^{\left(2\right)}=2n+N_{I}^{\left(2\right)}=\frac{1}{3}\left(2n^{3}-6n^{2}+13n-3\right), \end{array}$$

(A12) $$\begin{array}{cc} \; & D=1+2n+D_{I}=4n^{2}-14n+19. \end{array}$$

*Implementation via ancilla qubits*: A *k*-Toffoli gate, with k≥4, can be implemented using $N^{\left(a\right)}=k-3$ ancilla qubits and 4(k−3) Toffoli gates [62]. Accordingly, size and depth of this *k*-Toffoli gate are proportional to those of the Toffoli gate (k=3), which can be realized, e.g., with a linear-depth quantum circuit having $N_{\mathrm{Tof}}^{\left(1\right)}=0$, $N_{\mathrm{Tof}}^{\left(2\right)}=5$, and $D_{\mathrm{Tof}}=4$ (see previous paragraph) [61]. The increment gate (Figure 2b), in addition to one CNOT and one NOT, requires overall $1+\sum_{k=4}^{n}4\left(k-3\right)$ Toffoli gates (one standard Toffoli gate plus the decomposition of *k*-Toffoli gates with k=4,…,n), so it has $N_{I}^{\left(1\right)}=1$, $N_{I}^{\left(2\right)}=1+\left[1+\sum_{k=4}^{n}4\left(k-3\right)\right]N_{\mathrm{Tof}}^{\left(2\right)}$, and $D_{I}=1+1+\left[1+\sum_{k=4}^{n}4\left(k-3\right)\right]D_{\mathrm{Tof}}$. Therefore, the overall quantum circuit implementing the single time step of the DTQW (Figure 2d) has

(A13) $$\begin{array}{cc} \; & N^{\left(1\right)}=1+N_{I}^{\left(1\right)}=2, \end{array}$$

(A14) $$\begin{array}{cc} \; & N^{\left(2\right)}=2n+N_{I}^{\left(2\right)}=10n^{2}-48n+66, \end{array}$$

(A15) $$\begin{array}{cc} \; & D=1+2n+D_{I}=8n^{2}-38n+55, \end{array}$$

(A16) $$\begin{array}{cc} \; & N^{\left(a\right)}=\max_{k\in \{4,\ldots ,n\}}\left(k-3\right)=n-3. \end{array}$$

The number of ancilla qubits is independent of the number of time steps *t* and is determined by the *k*-Toffoli gate having the largest number of controls, as the ancilla qubits can be reused among the *k*-Toffoli gates of the increment gate [58].

*Implementation via rotations*—To conclude this section, we mention another scheme, introduced in [56], which uses rotations around the basis states to implement the increment gate. However, as pointed out by the authors, the number of gates needed by this scheme increases exponentially faster than for the ID scheme with ancillary qubits, although the latter scheme, as already discussed, quickly leads to a large workspace due to the number of required ancillary qubits which increases linearly with the number of control qubits. We do not delve into estimating $N^{\left(1\right)}$, $N^{\left(2\right)}$, and D in this scheme, so we refer the reader to [56] for the detailed computation of the (total) number of gates involved.

### Appendix C.2. QFT Scheme

We refer to the quantum circuit proposed in [58] as the QFT scheme (Figure 3). This scheme, based on the circuit design in Figure 2d, implements the increment gate by diagonalizing it via the QFT. In addition to the 2n CNOT gates (two-qubit gates, depth 2n; see remark (ii)) and the (I)QFT (see remark (iii)), the circuit requires a “layer” of *n*$R_{k}$ gates, which has unit depth (all the *n* one-qubit gates are executed in parallel). Therefore, the overall quantum circuit implementing the single time step of the DTQW (Figure 3) has

(A17) $$\begin{array}{cc} \; & N^{\left(1\right)}=1+2n+n=3n+1, \end{array}$$

(A18) $$\begin{array}{cc} \; & N^{\left(2\right)}=2n+2\frac{n(n-1)}{2}=n^{2}+n, \end{array}$$

(A19) $$\begin{array}{cc} \; & D=1+2n+2(2n-1)+1=6n. \end{array}$$

### Appendix C.3. Present Scheme

The quantum circuit proposed in the present work is shown in Figure 4. The (I)QFT is discussed in remark (iii). The “layer” of *n*$R_{k}^{†}$ gates and the coin gate have unit depth (all the n+1 one-qubit gates are executed in parallel). Then, the n−1 controlled-$R_{k}$ gates (two-qubit gates) amount to depth n−1 (sequential execution). Therefore, the overall quantum circuit implementing *t* time steps of the DTQW (Figure 4) has

(A20) $$\begin{array}{cc} \; & N^{\left(1\right)}=2n+t\left(n+1\right)=t\left(n+1\right)+2n, \end{array}$$

(A21) $$\begin{array}{cc} \; & N^{\left(2\right)}=2\frac{n(n-1)}{2}+t\left(n-1\right)=t\left(n-1\right)+n\left(n-1\right), \end{array}$$

(A22) $$\begin{array}{cc} \; & D=2(2n-1)+t(1+n-1)=tn+2(2n-1). \end{array}$$

These metrics comprise a fixed cost, due to the QFT and the IQFT, independent of the number *t* of time steps implemented, and a *t*-dependent cost. QFT and IQFT, unitary transformations, at the sides of the single time-step quantum circuit simplify each other when composing the circuit with itself.

## Appendix D. Transpilation of the Proposed Quantum Circuit for the Hadamard DTQW on the 4- and 8-Cycles

In the present work, we consider the Hadamard DTQWs on the 4- and 8-cycles described in Section 4.1. For these DTQWs, we implement the circuit proposed in Figure 4 on the IBM quantum computer ibm_cairo v.1.3.5, a 27-qubit Falcon r5.11 processor (qubit connectivity map in Figure 6a), whose native gates are

(A23) $$\begin{array}{cc} \; & CNOT=\left(\begin{array}{cccc} 1 & 0 & 0 & 0\\ 0 & 0 & 0 & 1\\ 0 & 0 & 1 & 0\\ 0 & 1 & 0 & 0 \end{array}\right),\;I=\left(\begin{array}{cc} 1 & 0\\ 0 & 1 \end{array}\right),\;R_{z}\left(\lambda \right)=\left(\begin{array}{cc} e^{-i\frac{\lambda }{2}} & 0\\ 0 & e^{i\frac{\lambda }{2}} \end{array}\right),\\ \; & \sqrt{X}=\frac{1}{2}\left(\begin{array}{cc} 1+i & 1-i\\ 1-i & 1+i \end{array}\right),\;X=\left(\begin{array}{cc} 0 & 1\\ 1 & 0 \end{array}\right). \end{array}$$

In Figure A1, we show the gate count—number of one- and two-qubit gates—and depth of the quantum circuit implementing *t* time steps of the DTQW on the 4- and 8-cycles, Figure A1a,b respectively, for optimization_level=1,3 in transpilation. Consistent with Table 1, the general trend is linear in time for both the DTQWs on the 4- and 8-cycles with light optimization (optimization_level=1) and only for the DTQW on the 8-cycle with the heaviest optimization (optimization_level=3). In the particular case of the 4-cycle, for the latter optimization level, the counts are basically independent of *t* (Figure A1a).

The transpiled circuit implementing one step of the Hadamard DTQW is shown in Figure A2 for the 4-cycle with optimization_level=1,3 and in Figure A3 for the 8-cycle with optimization_level=1. We recall that the initial state is given in Equation (23), the initial QFT is implemented via Hadamard gates (Appendix B), and the Hadamard gate, which is not a native gate in this quantum processor, is transpiled into $H=R_{z}\left(\pi /2\right)\sqrt{X}R_{z}\left(\pi /2\right)$ (see Equations (22) and (A23)).
